# αSMA Osteoprogenitor Cells Contribute to the Increase in Osteoblast Numbers in Response to Mechanical Loading

**DOI:** 10.1007/s00223-019-00624-y

**Published:** 2019-10-31

**Authors:** B. G. Matthews, N. K. Y. Wee, V. N. Widjaja, J. S. Price, I. Kalajzic, S. H. Windahl

**Affiliations:** 1 Department of Reconstructive Sciences, UConn Health, Farmington, CT, USA; 2 Department of Molecular Medicine and Pathology, University of Auckland, Auckland, New Zealand; 3 School of Veterinary Sciences, University of Bristol, Bristol, UK; 4 Royal Agricultural University, Cirencester, UK; 5 Division of Pathology, Department of Laboratory Medicine, Karolinska Institutet, Huddinge, Sweden

**Keywords:** Mouse, Axial loading, Lineage tracing, Osteoblasts, Osteoprogenitor

## Abstract

Bone is a dynamic tissue that site-specifically adapts to the load that it experiences. In response to increasing load, the cortical bone area is increased, mainly through enhanced periosteal bone formation. This increase in area is associated with an increase in the number of bone-forming osteoblasts; however, the origin of the cells involved remains unclear. Alpha-smooth muscle actin (αSMA) is a marker of early osteoprogenitor cells in the periosteum, and we hypothesized that the new osteoblasts that are activated by loading could originate from αSMA-expressing cells. Therefore, we used an in vivo fate-mapping approach in an established axial loading model to investigate the role of αSMA-expressing cells in the load-induced increase in osteoblasts. Histomorphometric analysis was applied to measure the number of cells of different origin on the periosteal surface in the most load-responsive region of the mouse tibia. A single loading session failed to increase the number of periosteal αSMA-expressing cells and osteoblasts. However, in response to multiple episodes of loading, the caudal, but not the cranial, periosteal surface was lined with an increased number of osteoblasts originating from αSMA-expressing cells 5 days after the initial loading session. The proportion of osteoblasts derived from αSMA-labeled progenitors increased by 70% (*p* < 0.05), and the proportion of αSMA-labeled cells that had differentiated into osteoblasts was doubled. We conclude that αSMA-expressing osteoprogenitors can differentiate and contribute to the increase in periosteal osteoblasts induced by mechanical loading in a site-specific manner.

## Introduction

Mechanical loading as a result of load-bearing physical activity is a major regulator of bone mass and microarchitecture [1, 2]. Load is sensed by bone cells as increased strain and results in modeling/remodeling processes that alter the bone architecture and form bones that are able to resist the habitual loads that are placed upon them. The increased bone formation is strain-dependent and site-specific; in response to axial loading of the mouse tibia, most bone is formed at the caudal periosteal site of the loaded bone [3–6]. Osteoblast numbers also increase following loading both in vivo [7, 8] and in vitro [7, 9]. However, the origin of the cells that contribute to new osteoblast formation in this setting is not well defined. The aim of this study was to assess the contribution of αSMA-labeled progenitors to osteoblast formation in the periosteum following mechanical loading.

We have recently shown that bone lining cells represent a major source of osteoblasts during adulthood [10], and lining cells contribute to new osteoblasts in various anabolic settings including following treatment with sclerostin inhibitors or PTH [11, 12]. Loading increases the number of periosteal osteoblasts in both male and female mice [7, 8]. However, the number of osteoblasts on an actively forming bone surface in response to load is approximately double that of the number of lining cells on a resting bone surface [8]. Since under most conditions, lining cells do not appear to proliferate following activation, osteoblasts must be recruited from cells other than lining cells in response to load [11, 12]. It has been suggested that loading and unloading affect the number of proliferative osteoprogenitor cells in that loading increases [8] and unloading decreases [13, 14] their proliferation and recruitment. However, the identity of these osteoprogenitor cells is not well defined.

We have identified alpha-smooth muscle actin (αSMA) as a marker of osteoprogenitor cells in bone [15–17]. These mesenchymal progenitors can differentiate into osteoblasts, adipocytes, and chondrocytes in vitro and in vivo and are located in perivascular niches, periosteum, and sutures. The fate of αSMA progenitor cells can be traced in vivo using tamoxifen-inducible αSMACreERT2 combined with a reporter. The αSMA-labeled cells in the periosteum and other tissues do not express markers of mature osteoblasts including transgenic Col2.3GFP a short period after labeling [15, 17, 18]. Using this model, we have demonstrated differentiation of tissue-resident αSMA-labeled cells in various settings. Specifically, αSMA-labeled cells contribute to osteoblasts and osteocytes during adolescent growth, cementoblasts, and periodontal ligament cells in the periodontium during growth and following injury, and odontoblasts following reparative dentinogenesis [15, 18, 19]. In the periosteum, αSMA-labeled cells are present in adulthood and contribute to osteoblasts, chondrocytes, and fibroblasts following fracture [15, 17].

In the present study, we have combined lineage tracing of αSMA-labeled cells, with a well-established marker of mature osteoblasts, Col2.3GFP [20], to track to origin of osteoblasts in the periosteum following an anabolic loading stimulus.

## Materials and Methods

### Mice

Procedures were approved by the UConn Health Institutional Animal Care and Use Committee and performed in an AAALAC accredited facility. Mice were housed in ventilated cages with a 12-h light cycle and temperature of 22 °C. Water and irradiated rodent chow (Teklad 2918, Invigo, Indianapolis, IN) was provided ad libitum. Male αSMACreER/Ai9/2.3GFP mice in a C57Bl/6J background were used for all studies and have been previously described [15, 20, 21].

### Mechanical Strain Measurements During Dynamic Axial Loading

**Fig. 1:**
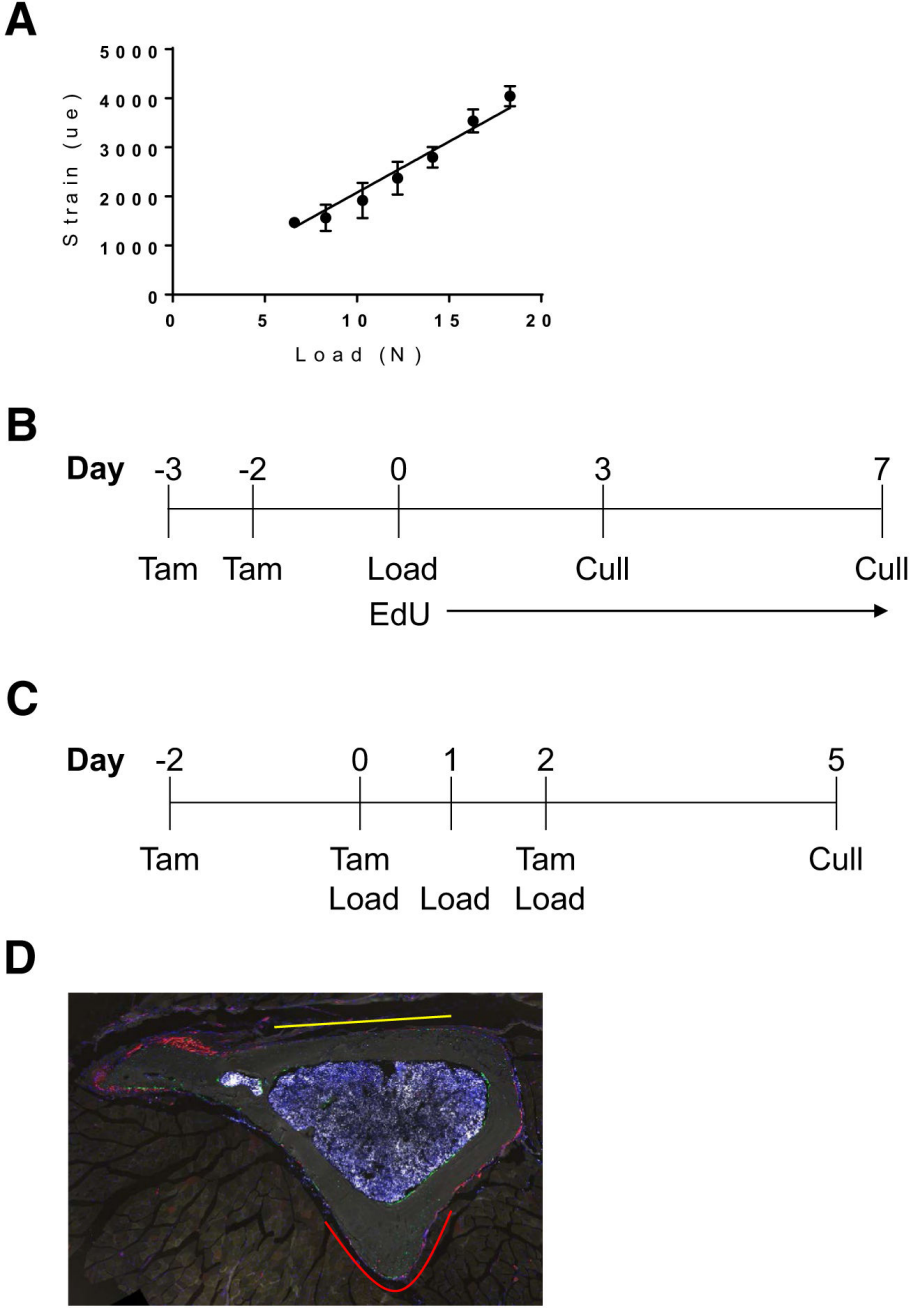
The load strain relationship and experimental outlines. **a** The load strain relationship shows that 2400 με was reached around 11.5 N. **b** Experimental design for the single loading experiment. EdU was injected twice daily. **c** Experimental design for the multiple episodes of loading experiment. **d** Tibial cross section indicating regions of the periosteum analyzed—the caudal region is indicated in red, and the cranial region in yellow.

The strain:load relationship in the tibiae of 4-month-old male C57BL/6 mice was performed as previously described [22, 23]. In short, strains were measured using a single element strain gauge (EA-06–015DJ-120, Vishay Measurement Group, PA, USA) at the medial aspect of the tibia at approximately 37% of its length from the proximal end across a range of peak compressive loads between 6 and 18 N (Fig. 1a). From the data, a peak strain corresponding to 2400 με was reached at 11.4–11.6 N.

### In Vivo Axial Loading of the Tibia

At 16–20 weeks of age, male mice were anaesthetized using isoflurane and their right tibiae were axially loaded. This involved positioning the tibia within the loading cups, securing in place, and applying 11.4 or 11.6 N (for the single and multiple episodes of loading, respectively) force at 1 Hz for 40 cycles, with a 10-s rest in between cycles using a 3100 ElectroForce® Test Instrument (TA Instruments, DE, USA) [22, 23]. The left tibia from the same mouse was used as a non-loaded control as previously validated [4, 5, 24].

### Experimental design

In order to stimulate new bone formation, loading is generally applied over a number of days; however, responses such as reduction in Sclerostin expression are evident 24 h after a single round of loading [7] and it has been suggested that a single loading is enough to promote endosteal and periosteal osteoblast proliferation in rats and roosters [8, 25]. We, therefore, divided the mice in two main groups receiving either a single session or multiple episodes of loading.

### Single loading

Tamoxifen (75ug/g of body weight; T5648; Sigma) was administered on days − 3 and − 2, then tibial loading performed on day 0. EdU (5-ethynyl-2′-deoxyuridine, 100 μl of 1 mg/ml solution) was administered twice daily to label proliferating cells. The animals were euthanized on days 3 (*N* = 6) and 7 (*N* = 5, Fig. 1b).

### Multiple Episodes of Loading

Two days prior to the study, male mice were pair-housed to limit natural physical activity within the cage yet maintain social interactions. Tamoxifen was administered on days − 2, 0, and 2, and tibial loading was performed on days 0, 1, and 2. The animals were euthanized on day 5 (*N* = 8) (Fig. 1c). Another cohort of animals were given tamoxifen (*N* = 4) or vehicle (corn oil *N* = 6) without undergoing the loading regimen.

### Tissue Collection, Sectioning, and Imaging

Mice were administered ketamine/xylazine cocktail and intracardiac perfusion was performed with PBS followed by 4% paraformaldehyde and fixed for 2 days. Tibiae were decalcified using 14% EDTA for 2–3 weeks, then transferred to 30% sucrose at 4 °C, prior to cryo-embedding with Cryomatrix (Thermo Fisher Scientific, Waltham, MA, USA). Bones were embedded to facilitate obtaining tibial cross sections, located approximately 37% from the proximal end of the tibia. Tissues were cut at 7 μm thickness on Cryotape (Cryofilm 2C, Section Lab, Japan) and cross-linked using Norland Optical Adhesive 61 (Norland Optical, Cranbury, NJ) onto glass slides. Sections were subsequently stained with Click-iT EdU Plus Imaging Kit, according to the manufacturer’s instructions (Alexa Fluor 647; Invitrogen). Slides were coverslipped in 50% glycerol with DAPI (1:5000, D1306; Molecular Probes). Slides were imaged using an Axioscan Z1 (Zeiss) with standardized settings maintained throughout each experiment.

### Image Analysis

For the image analysis, 3–6 sections of each tibia were analyzed. We targeted two regions for analysis, the caudal and cranial surfaces, which sense compressive and tensile strain, respectively, during loading (Fig. 1d). In response to axial mechanical loading, bone formation is induced at both these sites, but the response is stronger at the caudal than the cranial site [3, 4, 26]. For the single loading study, nuclei were counted manually in the ROIs using Zen software (Zeiss), followed by determination of the number of cells expressing one or more fluorescent labels. For the multiple episodes of loading study, analysis was performed using ImageJ2 software (NIH, Bethesda, MD, USA). The DAPI channel underwent background subtraction followed by thresholding using the Li method and segregation by the watershed function. Particle analysis was performed and nuclei were counted based on size (10–150 μm^2^) and circularity (> 0.5) within the ROIs, and signals in the red and green channels measured. Thresholds were determined manually and applied in Excel. The length of the bone surface analyzed was also measured. Osteoblast counts were determined by dividing the number of GFP+ cells by the length of the surface evaluated, αSMA numbers were calculated in a similar manner using the number of tdTomato+ cells. For αSMA differentiation, the number of dual positive GFP+ tdTomato+ cells was divided by total tdTomato+ cells. For osteoblasts derived from αSMA+ cells, the number of dual positive cells was divided by total GFP+ cells.

### Statistical Analyses

Statistical analysis was performed in GraphPad Prism 7. Normality tests suggested that the datasets were not normally distributed so Wilcoxon matched-pairs signed rank tests were performed. The proportion of osteoblasts derived from αSMA+ cells in the caudal region was considered the primary outcome, where *p* < 0.05 is statistically significant. For other comparisons, a Bonferonni correction was applied so the adjusted significance threshold was *p* < 0.00714. Full p value results for the multiple loading experiment are shown in Table 1.

## Results

The effect of tamoxifen on osteoblast numbers was tested following three injections of tamoxifen. We did not find any difference in the number of osteoblasts per bone surface in either the cranial or caudal sites (Supplemental Fig. 1).

### A Single Session of Intermittent Loading Does Not Enhance the Number of Periosteal αSMA+ Cells or Osteoblasts

**Fig. 2:**
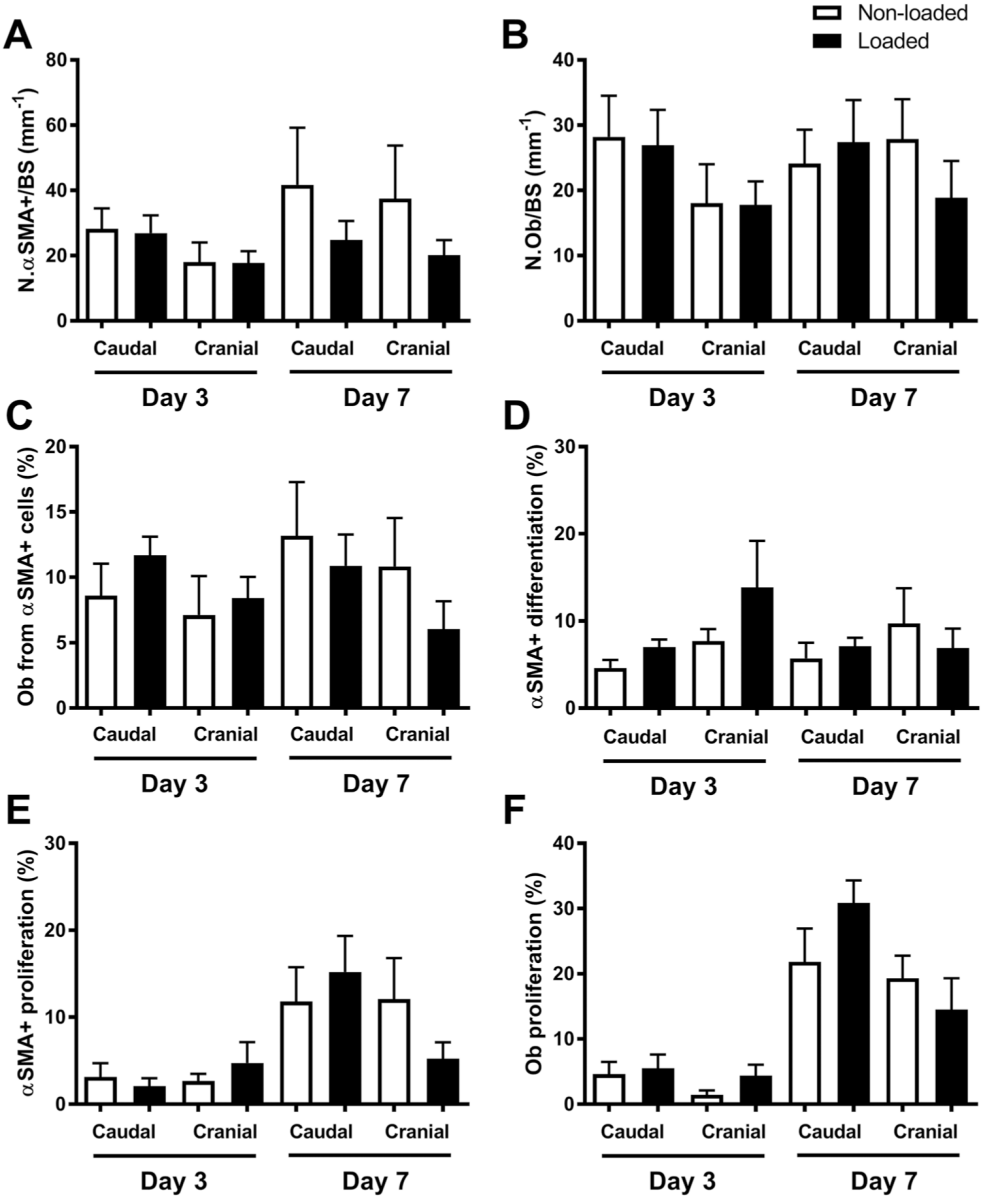
A single session of intermittent loading does not enhance the number of periosteal αSMA+ cells or osteoblasts (Ob). **a** αSMA-labeled cell numbers and **b** GFP+ osteoblast numbers per bone surface in the periosteum in the indicated regions. **c** The percentage of osteoblasts derived from αSMA-expressing cells and **d** the percentage of αSMA+ cells that differentiated into osteoblasts. **e** The percentage of αSMA-labeled cells that proliferated following loading and **f** GFP+ osteoblasts that proliferated following loading. Bars show mean ± SEM, *n* = 6 for day 3, *n* = 5 for day 7. *p < 0.05

**Fig. 3:**
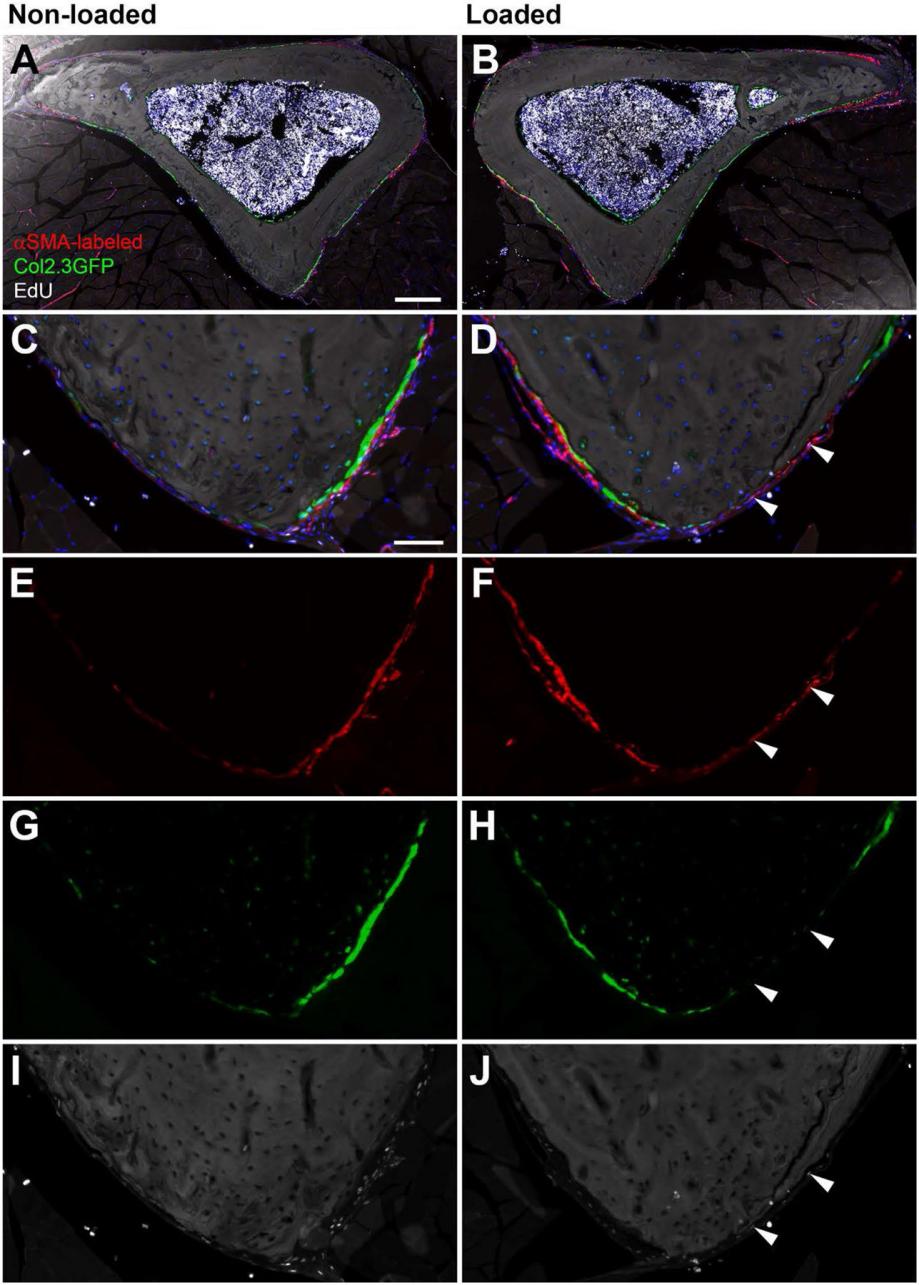
Representative images of non-loaded (**a**, **c**, **e**, **g**, and **i**) and loaded (**b**, **d**, **f**, **h**, and **j**) bones. The caudal region is magnified in **c**–**j**. Overview of the tibia cross section (**a** and **b**), αSMA+ cells in red (**e** and **f**), Col2.3GFP+ osteoblasts in green (**g** and **h**), and EdU+ cells in white (**i** and **j**). DAPI staining is shown in blue. Scale bars represent 200 μm (**a**, **b**) or 50 μm (**c**, **j**). Arrowheads indicate αSMA+ EdU+ cells in the caudal region

We initially evaluated the effect of a single round of axial loading on the number of Col2.3GFP+ osteoblasts, αSMA-labeled cells, and proliferating cells. We were unable to detect any difference in parameters associated with a bone formation response to loading either 3 or 7 days following loading. Specifically, there was no increase in the number of αSMA-labeled cells or GFP+ osteoblasts in the loaded limb (Fig. 2a, b), nor was there any change in the contribution of αSMA-labeled cells to osteoblasts with response to loading or in relation to the time since loading and labeling (Fig. 2c, d). While we observed EdU incorporation into a subset of periosteal cells, including both αSMA-labeled cells and osteoblasts, at the time points investigated loading did not significantly change proliferation in the regions analyzed (Figs. 2e, f and 3).

### Multiple Episodes of Axial Loading of the Tibia Enhances the Number of Differentiating αSMA‑Positive Cells and Osteoblasts Derived from αSMA+ Cells at the Caudal Periosteal Site

**Fig. 4:**
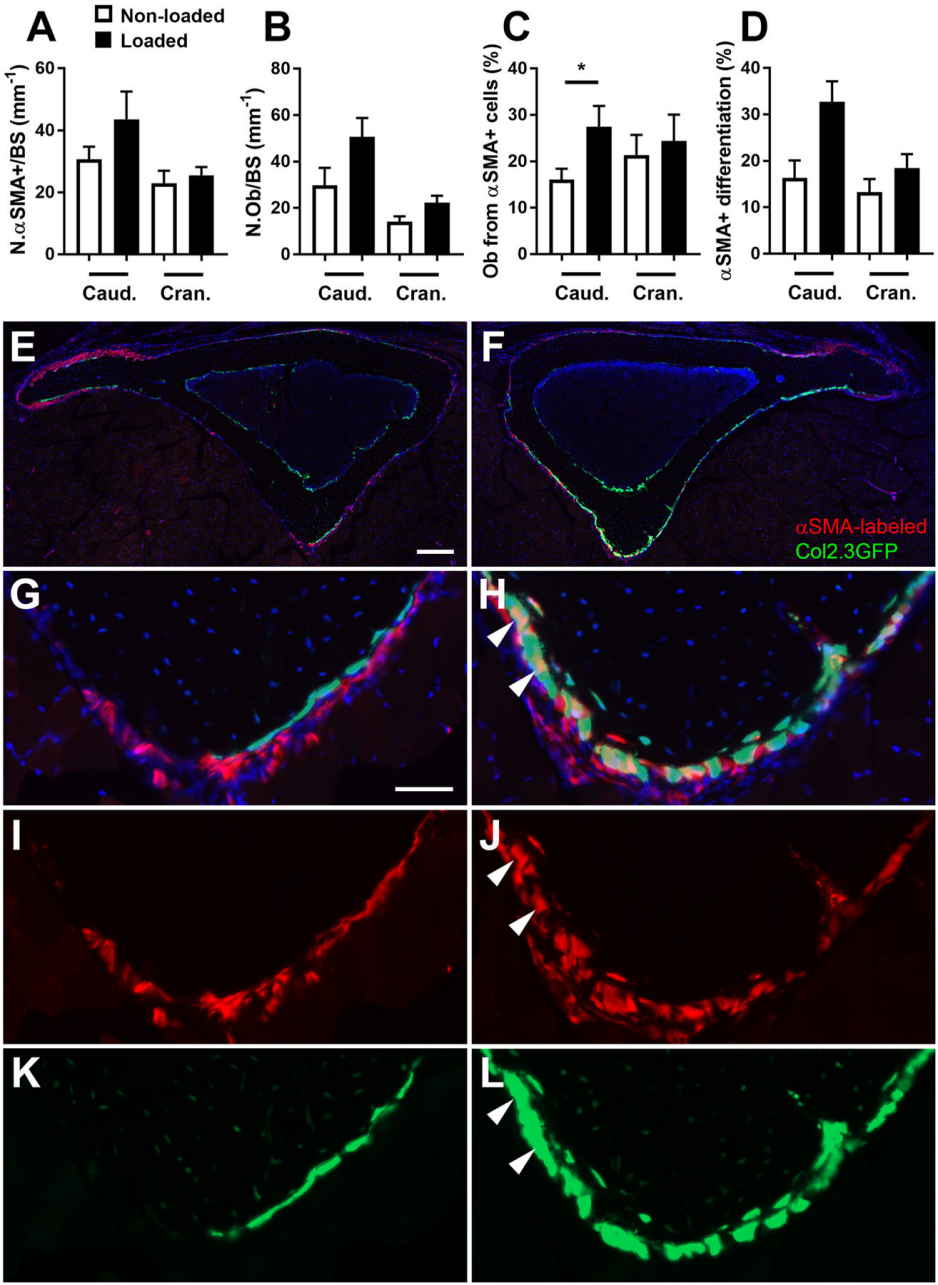
Axial loading of the tibia enhances differentiation of periosteal αSMA+ cells into osteoblasts (Ob) after multiple episodes of loading. **a** αSMA-labeled cell numbers and **b** GFP+ osteoblast numbers per bone surface in the periosteum in the indicated regions. **c** The percentage of osteoblasts derived from αSMA-expressing cells and **d** the percentage of αSMA+ cells that differentiated into osteoblasts. Representative images of non-loaded (**e**, **g**, **i,** and **k**) and loaded (**f**, **h**, **j**, and **l**) bones are shown with the caudal region magnified in **g**–**l**. DAPI staining is depicted in blue. Bars show mean ± SEM, *n* = 8. * statistically significant (see methods for p value thresholds). Scale bars represent 200 μm (**e**, **f**) or 50 μm (**g**–**l**). Arrowheads indicate selected αSMA+ Col2.3GFP+ cells.

In order to enhance the bone formation response to mechanical loading, we loaded tibiae on three consecutive days. Five days after initiating multiple episodes of loading, we did not detect any difference in the nuclei/μm (data not shown) or in the number of αSMA+ cells at either the caudal or cranial sites (Fig. 4a, Table 1). The number of GFP+ osteoblasts increased by 58% at the cranial site. At the caudal site, the change was not statistically significant, although 6/8 animals showed increased osteoblast numbers, representing more than doubling of the osteoblast numbers on average in the caudal site of the loaded limb (Fig. 4b, e–l). However, differentiation of αSMA-labeled progenitor cells into osteoblasts was significantly enhanced in the caudal region. We saw both a 70% increase in the proportion of osteoblasts derived from αSMA-labeled progenitors (Fig. 4c) and a doubling in the proportion of αSMA-labeled cells that had differentiated into osteoblasts at the caudal site (Fig. 4d). Contribution of αSMA-labeled cells to osteoblasts did not increase in the cranial region.

## Discussion

Mechanical loading causes a rapid anabolic response in specific regions of the periosteum [3–6]. However, the origin of the cells that contribute to new osteoblast formation in this setting is not well defined. In this study, we assessed the contribution of αSMA-labeled progenitors to osteoblast formation following mechanical loading. We have previously demonstrated that αSMACreERT2 labels a population of progenitors in the periosteum that make a major contribution to both osteoblasts and chondrocytes within the fracture callus, but does not initially identify osteoblasts [15, 17].

The bone anabolic response to loading is site-specific, where most bone formation is seen at the periosteal caudal site [3, 26]. Therefore, we investigated the number of osteoblasts and their origin at the periosteal caudal site and compared it to the cranial surface where less bone is formed in response to load. We were unable to see evidence of enhanced osteoblast proliferation or differentiation of osteoprogenitors into osteoblasts with a single episode of loading. This is in contrast with previous work in rats and roosters where a single round of loading promoted an increase in osteoblast numbers and bone formation 3–5 days after loading [8, 25]. The apparent discrepancy could be related to differences in sex, strain applied, or species differences. Although the study in rats did not report the strains applied, the study in roosters applied strains of 2700–3000 μ strain [25], which is higher than the strains used in this study.

In contrast to single loading, multiple episodes of loading enhanced the osteoblast number in the periosteum in 6/8 animals. This is in line with a previous study showing that the periosteal bone-forming surface increases in a dose-dependent manner with increasing number of loading sessions [27]. The authors of that study proposed that a quantum of bone cells is activated in response to each loading event, whereas the strain magnitude dictates the size or microstructural organization of each quantum of bone cells.

Following multiple episodes of loading, while osteoblast number did not increase in all samples in the caudal region where most bone is formed in response to load [3, 26], we consistently saw increased contribution of αSMA+ progenitors to the osteoblast pool, indicating that loading promoted increased differentiation of these cells. This was not seen in the cranial region where less load response is seen. The mechanisms whereby load stimulates differentiation of αSMA+ progenitors are unknown, although they are likely to involve osteocytes sensing the increasing strain in the bone and then communicating with the periosteal αSMA+ progenitors via signaling molecule/s. We have demonstrated previously that αSMA labels periosteal-resident cells in adult mice [15, 17]. These are a mixed population of cells, but a subset expresses mesenchymal stem and progenitor markers such as Sca1. We cannot completely exclude contribution of skeletal muscle-derived cells as αSMA does label both myogenic and mesenchymal populations in the muscle [28]. However, studies of fracture healing indicate that muscle cells can act as chondroprogenitors, but only contribute to osteoblasts within the fracture callus when the periosteum is extensively damaged or removed [29, 30]. On this basis, we believe it is highly unlikely that muscle-resident cells are contributing to osteoblast formation following loading. Studies using bone-restricted markers like Osterix during loading appear to confirm this [31].

It is difficult to estimate the exact contribution of the αSMA+ progenitor population to loading-based bone formation for a number of reasons. Firstly, most of the surfaces analyzed, even those from non-loaded bones, had Col2.3GFP+ osteoblasts present. The lifespan of osteoblasts in vivo is difficult to define, but recent studies suggest around 2 weeks is a reasonable estimate in adult mice [10, 32]. Therefore, some of the osteoblasts on the surfaces analyzed were likely present prior to tamoxifen delivery, meaning they could not have been αSMA-labeled. Assuming the non-loaded leg represents the number of ‘old’ osteoblasts, we estimate that an average of 52% of ‘new’ osteoblasts is derived from αSMA+ progenitors. However, given there are osteoblasts derived from αSMA-progenitors in the unloaded limbs as well, new osteoblast formation was clearly also occurring on these surfaces under both loading regimens. In addition, the efficiency of the inducible Cre recombination is rarely 100%. Indeed, αSMACreER labels about 50% of αSMA-expressing cells when analyzed in vitro (BGM, IK in preparation). Thus, it is difficult to estimate exactly what proportion of the newly recruited osteoblasts are originating from αSMA osteoprogenitors, but our numbers suggest that there is another source of osteoblasts outside the αSMA lineage. A recent study showed that genetic ablation of Prx1-CreER cells, which likely represent a similar, or at least overlapping subset of periosteal progenitors, significantly reduced bone formation rate in response to ulnar loading [33]. Although reduced, the anabolic response to loading was not completely lost, despite evidence that ablation was close to 100% effective, supporting the idea that a non-progenitor population such as bone lining cells also contributes to the loading response. It was also recently reported that osterix (Osx) lineage cells identified in adulthood using Osx-CreER contribute to most or all of the bone surface cells associated with mineralization within the days following loading in a similar model [31]. Osx is expressed throughout the osteoblast lineage including in progenitors, osteoblasts, and bone lining cells, and we have previously demonstrated Osx expression in αSMA+ periosteal cells [10, 17]. These results are therefore consistent with our conclusion that αSMA+ progenitors contribute to some osteoblasts while others likely arise from more differentiated cells within the lineage that no longer express αSMA.

Our study has some limitations in that we were not able to show enhanced osteoblast proliferation or osteoprogenitor differentiation into osteoblasts with a single round of loading. It is possible that if we had used a higher strain magnitude, we may have been able to detect a significant increase in osteoblast differentiation and/or number. However, a higher strain may not reflect the strains that are normally placed upon the skeleton. Another limitation of this study is that we induced the expression of the reporter transgene by tamoxifen. Tamoxifen is a selective estrogen receptor modulator known to independently enhance bone formation and to modify the loading response at concentrations at or below what is used for most lineage tracing studies [24]. However, in agreement with Zannit and Silva [31], we could not detect any statistically significant difference in osteoblast number per bone surface when comparing vehicle and tamoxifentreated mice. While some osteoblasts may have been present prior to tamoxifen or loading, our results indicate that another population in addition to αSMA-labeled cells contributes to osteoblasts in this scenario.

In conclusion, this study has demonstrated that mechanical loading increases the differentiation of osteoblasts from αSMA-labeled progenitors at specific sites on the periosteal surface.

## Supplementary Material

Supplemental Figure

## Figures and Tables

**Table 1:** *P* values associate with Wilcoxon matched-pairs signed rank test for multiple loading parameters (Fig. 4)

	N.αSMA+/BS	N.Ob/BS	αSMA+ differentiation	Ob from αSMA+ cells
Caudal L vs R	0.1953	0.1484	0.0391	0.0156*
Cranial L vs R	0.6406	0.0078	0.3125	0.6719

* Primary outcome, therefore *p* < 0.05 is statistically significant. Significant *p* value for other parameters following Bonferonni correction is *p* < 0.00714
