# Supplementary figures and images for: αSMA Osteoprogenitor Cells Contribute to the Increase in Osteoblast Numbers in Response to Mechanical Loading
Source: Calcif Tissue Int. Author manuscript; Available in PMC 2020 Feb 3. (PMC6995756; doi:10.1007/s00223-019-00624-y)

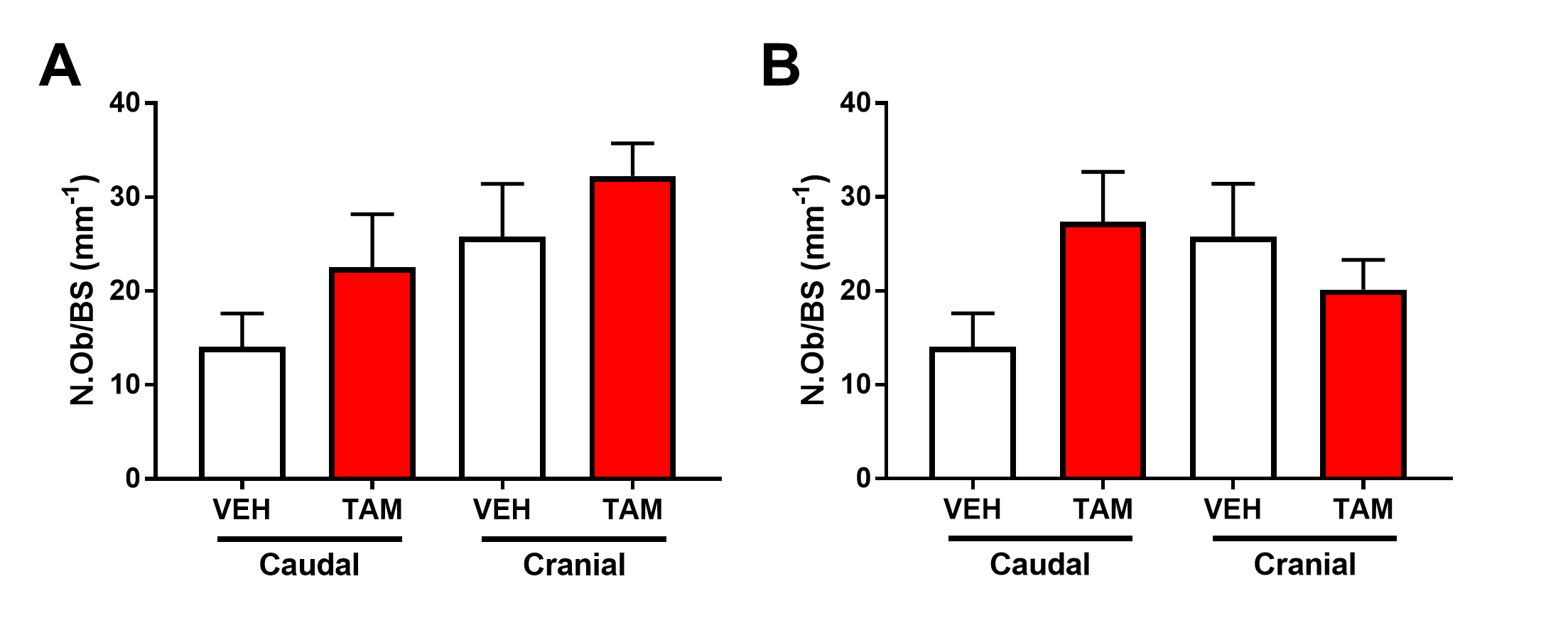

Supplement: Supplemental Figure [file NIHMS1058905-supplement-Supplemental_Figure.tif]
